# Reflections on the implementation of the Italian guidelines on comprehensive geriatric assesment for the older persons

**DOI:** 10.1007/s40520-025-03036-1

**Published:** 2025-05-12

**Authors:** Alberto Castagna, Giovanni Ruotolo, Margherita Azzini, Pierangelo Lora Aprile, Nicola Vanacore, Alberto Pilotto, Nicola Veronese

**Affiliations:** 1 SOC Cure Primarie, Distretto di Soverato, Azienda Sanitaria Provinciale Di Catanzaro, Catanzaro, Italy; 2 Department of Medical and Surgical Science, University Magna Grecia, Catanzaro, Italy; 3 SOC Di Geriatria, AOU “ Renato Dulbecco”, Catanzaro, Italy; 4 UOC Di Geriatria, AULSS9 Scaligera Verona IT, Verona, Italy; 5 College of General Practitioners and Primary Care, Florence, Italy; 6 National Center for Disease Prevention and Health Promotion, Italian National Institute of Health, Rome, Italy; 7 Department of Geriatric Care, Neurology and Rehabilitation, Galliera Hospitals, Genoa, Italy; 8 Department of Interdisciplinary Medicine, “Aldo Moro”University of Bari, Bari, Italy; 9 Faculty of Medicine, Saint Camillus International, University of Health Sciences, Rome, Italy; 10 Geriatrics Section, Department of Internal Medicine, University of Palermo, Palermo, Italy

**Keywords:** Comprehensive geriatric assessment, Guidelines, Implementation

## Abstract

A fundamental phase in the life of new Guidelines is the implementation, which responds to the need to facilitate their adoption with interventions and procedures of proven effectiveness to increase their impact on public health. The implementation should follow a scientific method, and therefore shares many characteristics and the rigorous approach of clinical research. However, it differs in purposes, methods, and aims by addressing factors that include identifying and resolving barriers and facilitators in the adoption of evidence-based clinical innovations. The use of comprehensive geriatric assessment (CGA) can be facilitated by technological tools, with the possibility of early diagnosis of several chronic conditions, monitoring and management of the diseases typical of older people and, finally, to personalized care and optimization of healthcare resources. However, remote CGA also has limitations, including technology requirements, data security/privacy, and the need for comprehensive evaluation and simplicity. In this document we present the history and the model of implementation of the Italian guidelines on CGA in older persons. The standardization of CGA in older adults across different settings is particularly important in countries like Italy, that have among the oldest world population and where broader implementation of CGA, also outside traditional geriatric settings, has become a health priority that cannot longer be delayed.

## Introduction

The Italian guidelines on comprehensive geriatric assessment (CGA) for the older persons arise from the need to define, on the basis of the evidence in the literature, the scientific, conceptual and procedural bases for the application of CGA in the different clinical contexts (general medicine and primary care, hospital area, residential area, palliative care area) in which a multidimensional and interdisciplinary assessment and treatment path is required for the older person, particularly in terms of care, assistance and rehabilitation [1]. For this reason, the promoters of this Guidelines, i.e., the Italian Society of Geriatrics Hospital and Territory (SIGOT) and the Italian Society of General Medicine and Primary Care (SIMG), with the National Institute of Health (in Italian Istituto Superiore di Sanità (ISS) as the methodological guarantor of the entire production process of the Guidelines, have decided to immediately share this path with the widest possible number of professionals in the social and health area. After the publication of the document in English [1], an essential phase of the life cycle of the Guidelines, in which they produce value in the National Health System, is the Implementation. Once the guidelines have been produced, aspects of their implementation remain critical. The literature exploring the time lags between early research and possible translation into health improvements is less extensive than that which focuses specifically on the diffusion of innovations [2]. The time taken, or time lag, between the publication of a work and its application in clinical practice with interventions that lead to health and broader benefits is a topic of increasing interest and investment by stakeholders to maximize the returns of such papers. Implementation is the moment in which this tool reaches its final purpose, that of “influencing” the decision-making process [3]. This process consists of changing individual and collective behaviors so as to reduce unwanted variability in clinical practice and continue in the direction suggested by the guidelines with the aim of obtaining an improvement in clinical outcomes [3]. An important aspect of the Italian guidelines on CGA for the older persons was the publication of the document on the website of the National Guidelines System guidelines that further underlines the high transparency and the methodological values of this work.

### Diffusion of innovations

In his seminal work on “Diffusion of Innovations”, published in 1962, Everrett Rogers first conceptualized the diffusion of innovation as a social process with multiple determinants, understood as *“the process by which an innovation is communicated through certain channels over time among the members of a social system”* [4]*.* The theory focuses on the speed with which different individuals, within a social system, adopt an innovation (adoption rate) [4]. The Diffusion of Innovations represents the process through which an individual moves from first knowledge of an innovation towards forming an attitude to it, to a decision to adopt or reject it, to implementation of the new idea, and to confirmation of this decision. The innovation decision process includes five phases (Table 1) [5, 6].

**Table 1:** Phases of the innovation decision process

1	Knowledge, when the individual is exposed to the innovation’s presence and understands how it works
2	Persuasion, when the individual creates a favourable or unfavourable attitude towards the innovation
3	Decision, when the individual gets engaged in activities that result in a choice to adopt or reject the innovation
4	Implementation, when the individual puts an innovation to use
5	Confirmation, when the individual seeks reinforcement for an innovation-decision already made, but may reverse the decision, if exposed to conflicting messages about it

The adoption of an idea usually occurs in an S-shaped curve. [5, 6] More specifically, the adoption distribution exhibits an S-shaped curve over time and approaches normality. In fact, an innovation is firstly adopted by a few individuals or firms. As more use it, others observe its use, and if the innovation is better than what went previously, others start to adopt and use it. When the diffusion reaches a level of critical mass, it proceeds faster. The critical mass takes place at the point at which enough individuals in a system have adopted an innovation; therefore, the innovation’s further rate of adoption becomes self-sustaining [5, 6]. Accordingly, it is based on such adoption behaviours that the S-curve and bell-shape curve are developed, and that grouped the adopters. In particular, there is a typical shape for a diffusion curve when innovations are developed successfully and stay undisturbed in a social system [6]. At the outset, the adoption rate is low, but it then increases gradually and decreases again towards the end. If it is presented graphically as a curve of percentages, it normally takes the form of an S-curve [6]. If the rates of adoption are taken as an absolute number of adopters per unit of time rather than in percentages, the outcome is a bell-shaped or wave curve, similar to a normal distribution [6]. Classified according to the rates of adoption of innovations, the adopter categories represent the classifications of the members of a social system in relation to the level to which an individual or other unit of adoption is relatively earlier in adopting new ideas in comparison to other members of a system [6]. These five adopter categories are the innovators, early adopters, early majority, late majority and laggards [6]. If the rates of adoption are taken as an absolute number of adopters per unit of time rather than in percentages, the outcome is a bell-shaped or wave curve, similar to a normal distribution [6]. Early Knowers of an innovation are generally a higher qualification. Early adopters are also different from late adopters in terms of personality factors [7]. They have more empathy, less dogmatism, a greater ability to deal with abstractions, greater rationality, higher intelligence, greater self-efficacy, higher aspirations for formal education, and higher-status roles [7]. It is worth noting that the distinct characteristics of the five adopter categories indicate that these adopter categories can be helpful in audience segmentation, a strategy in which several communication channels and/or messages are referred to reach each sub-audience [6]. According to Rogers’ *model of diffusion of innovations, in general, then, “innovations perceived as having more advantages, compatibility, ability to be tested, observability and less complexity will be adopted more quickly*” [5]. Other authors have underlined relevant contextual aspects, such as the use of knowledge (knowledge utilization) or technological transfer (technology transfer), which does not proceed freely and autonomously even within individual organizations [8]. The Implementation Process can be divided into three pillars [9]. The first pillar is represented by training and communication activities, both towards professionals and patients. The second pillar consists of the structuring of the guidelines and their transfer to clinical practice through the appropriate tools (networks, diagnostic and therapeutic care pathways, i.e., PDTA, multidisciplinary teams, ICT infrastructures). The third and final pillar is made up of application, i.e., guidelines should generate teamwork, patient engagement and leadership styles. Process elements that should not be overlooked, according to this structure, lie in the need to align implementation and update times, the involvement of stakeholders and the formulation of appropriate monitoring indicators [1]. Implementation normally consists of different tasks, as reported, in our case, in the Gantt chart (Table 2). A visual overview helps you make sure that everything is in the right place on the timeline and nothing is forgotten. Task names are normally set on the Gantt chart. Milestones are the little “*wins*” of the projects. They are normally at the end of the task and hold some significance for the project. Usually, milestones are displayed as diamond-shaped, which looks like a bonus after the completion of the task. As illustrated in the Gantt Chart (Table 2), we are still in the training and communication activities phase, carried out not only with local, regional and national conference events, but also in international events. Of particular interest are the training courses on the use of specific CGA tools. In some local areas, the structuring of the use of the indicated CGA has begun, with pilot experiences that will be analysed for the correct evaluation.

**Table 2:**
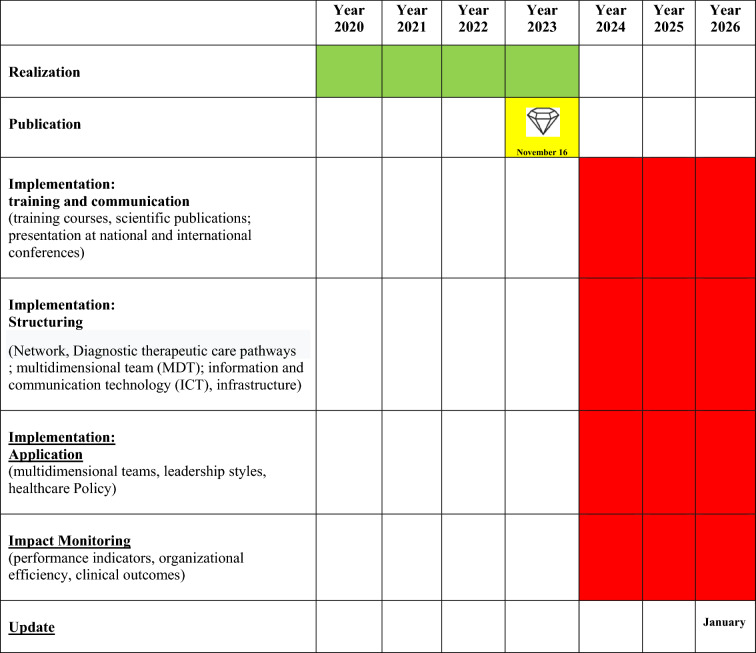
Gantt chart

The Italian guidelines on CGA for the older persons, also contain general indications on their implementation and dissemination as well as the forecast of the update date (January 2026) in consideration of the continuous evolution of medical-scientific knowledge [6]. Multiple ways of disseminating of the document have already started (Table 3).

**Table 3:** Ways of disseminating of Italian guidelines on CGA in older persons

Dissemination in media and press
Mail shipment to reference centers
Publication on the SNLG-ISS website (November 2023; link: https://www. iss. it/-/valutazione- multi dimensionalpersona- anziana- 1.)
Publication on the websites of scientific societies, health agencies, etc
Scientific publications
Presentation at national and international conferences

Experience shows that providing general indications of the implementation process is not in itself sufficient to guarantee automatic assimilation into clinical practice, especially in cases where, like our guidelines, they do not concern only one discipline (Geriatrics), but several other medical and non-medical disciplines. In other words, with this document, we want to maximize the generalizability of the implementation process of the Italian guidelines on CGA for older persons in heterogeneous settings, which, due to the complexity of the patient, should have a common language. As with all implementation models, it was necessary to identify possible facilitating and hindering factors. When considering factors facilitating the clinical and organizational application of this Guideline, scientific literature suggests some important factors. First, to develop training and educational programs specifically including information on the utility and operational methods of CGA [1, 10]. This Guideline suggests, based on scientific literature, multidimensional tools and their most appropriate and useful application for patients, caregivers, and health and social care professionals. Moreover, it is essential to promote, at local level, CGA integrated into specific pathways compatible with individual territorial entities. Widening the communication skills of the health and social care professionals included as target population in the Guidelines to facilitate the implementation of multidimensional assessment paths and the planning of individualized care programs [1]. In our view, once a potential model is established, the CGA remains crucial in determining the optimal timing for care interventions [11, 12]. Among the possible obstacles to the dissemination and implementation of this Guidelines, the need for a digital computerization of these tools should be mentioned to overcome the current heterogeneity of multidimensional tools, and to facilitate the sharing of information and shorten the CGA procedures [13]. The need for organizational adaptations should also be considered, including the acquisition of trained and dedicated personnel in the different settings (hospital, territorial and residential areas) that the application of the recommendations of this Guideline may require.

### The implementation of the CGA in the new Italian legislative context

As regards Italy, the implementation of guidelines on the CGA for the older persons must be carried out from the perspective of Law n. 33 of March 2023 (Gazzetta Ufficiale n. 76 of 30/03/2023). One of the most relevant parts of the same is certainly that of the provision of a unified multidimensional assessment, to be carried out according to standardized and homogeneous criteria based on guidelines validated at national level. The guidelines wanted to identify the bio-psycho-social, socio-health and health needs of the older person and their family unit and ascertaining the main conditions for access to state-responsible services. Finally, this work, wants also to take into account any information in possession of the third sector providing the services, intended to replace the procedures for assessing civil disability and the conditions for accessing benefits used today.

### SWOT analysis

**Fig. 1:**
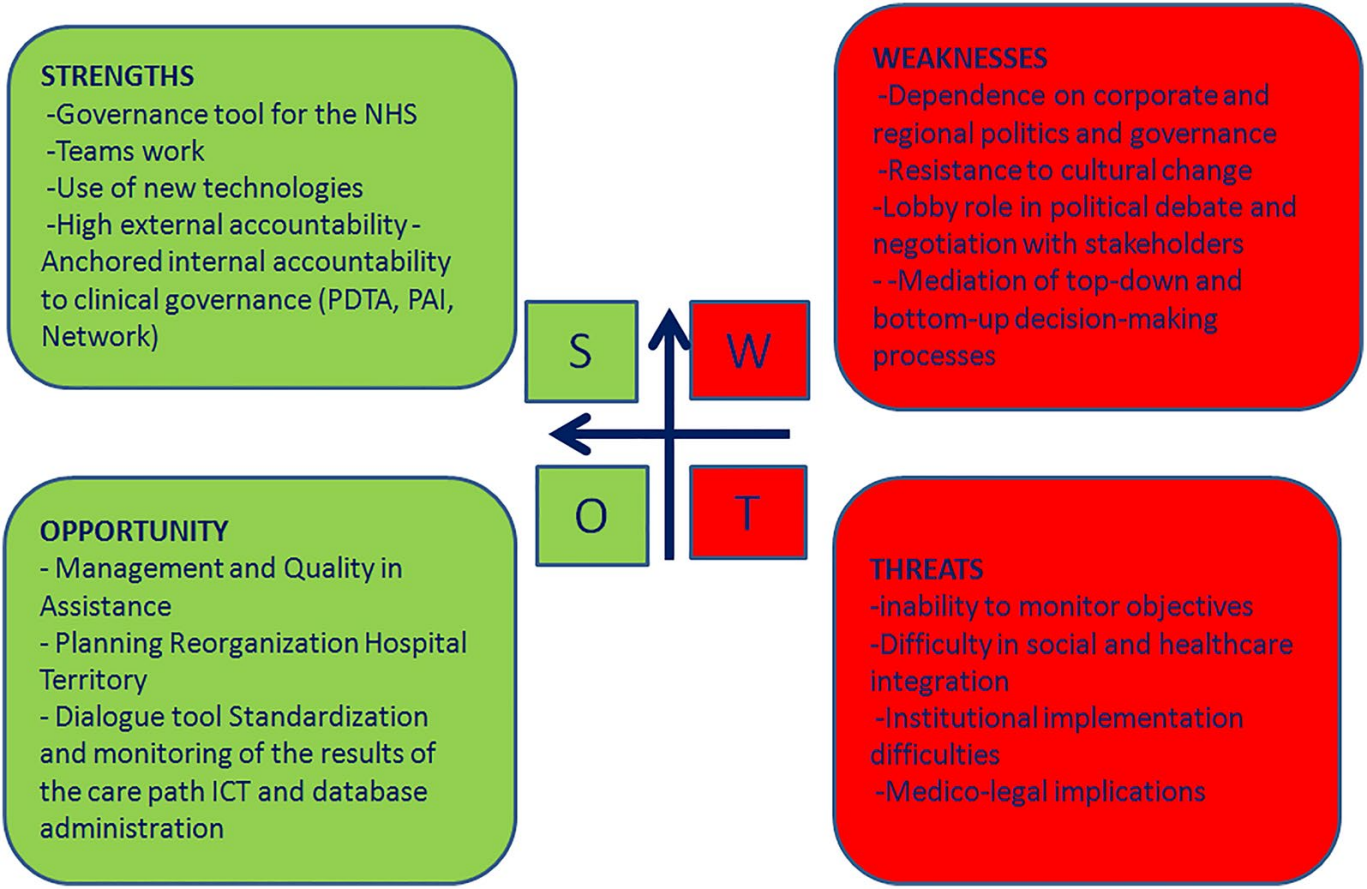
SWOT analysis

SWOT (Strengths, Weaknesses, Opportunities, Threats) Analysis is a business strategy tool to assess how an organization compares to its competition. The strategy is historically credited to Albert Humphrey in the 1960 s, but this attribution remains debatable [14]. There is no universally-accepted creator [15]. The complete set of positive (benefits) and negative (risks) effects expected from the implementation of the Italian guidelines on CGA for the older persons can be examined synthetically with the support of a SWOT matrix which provides a key quick and intuitive reading. The SWOT analysis is a strategic planning tool used to evaluate several aspects of a project or in any other situation in which a organization or an individual must make a decision to achieve an objective. The analysis can concern the internal environment (analyzing strengths and weaknesses) or external environment of an organization (analyzing threats and opportunities). The representation of this type of analysis is shown in Fig. 1. The SWOT analysis, especially with a view to a national management, can only be considered useful if applicable in individual regional realities. This is why our Teams carried it out as an examination of the areas that could benefit from an improvement in the general leadership style and in the Stewardship model of the Ministry of Health on the Regional Health Departments and consequently also on the local Health Authorities. Although national legislation may appear to be a limitation, the goal of our team is to provide a universal geriatric medicine thinking tool. Indeed, despite countries having different development backgrounds, the goal of defining a content of targeted education and training activities for healthcare professionals in various clinical contexts, adapted to the local context, appears important. A constructive comparison can be observed in the context of COST PROGRAMMING (PROmoting GeRiAtric Medicine IN countries where it is still eMerGing, https://cost-programming.eu), an important initiative supported by several European institutions and aimed at advancing geriatric medicine in countries where it is not yet well-established. The goal is to support the development of geriatric care through education, training, advocacy, and collaboration, helping these countries build capacity to address the needs of aging populations.

### Performance indicators

The expected result of the implementation process of the Italian guidelines on CGA for the older persons is greater appropriateness and interdisciplinary, socio-health integration, a dialogue between the various hospital and territorial care settings, thanks to the possibility of dialogue in a personalized way of the needs of the patient and their caregivers [1]. Another expected result is the increase in personalized assistance, from prevention to treatment, also thanks to the use of telemedicine thanks to the possibility of an intra-setting dialogue necessary to manage the grey areas present between the various hospital, residential and home settings [16]. The stronger the dialogue, the better and more effective the systems for taking charge of the most highly complex situations and the lower the total costs on the healthcare system [17]. The performance indicators must produce information aimed at supporting decision-making processes, measuring, detecting and communicating company results as well as monitoring the objectives present in the reorganization project [17]. The following monitoring indicators are proposed:

- Number of specific training events organized by scientific societies;
- Proportion of older patients assessed with a multi-dimensional approach in the different care settings assessed in at least three territorial areas of the North, Center and South of the Islands;
- Change in outcomes such as mortality rate, emergency room access rate or hospital admission rate assessed in at least three regions of the North, Central and South Islands [1].

### Reflections on the implementation

**Fig. 2:**
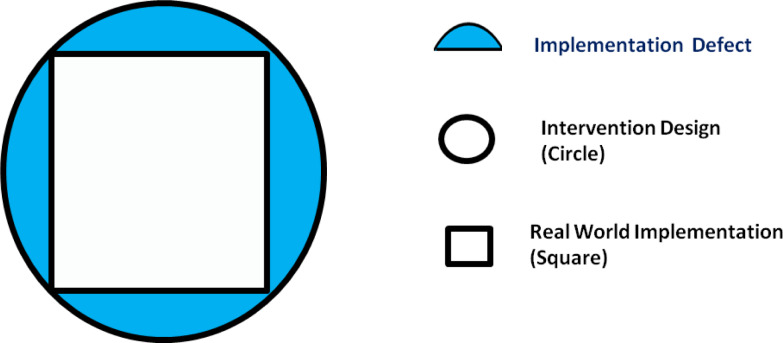
Squaring the circle (the air in blue represents the component of the project that was not implemented. This reflects the gap between the intervention as it was versus the intervention as it was conceived or planned)

The implementation of the CGA Guidelines requires the use of a series of disciplines ranging from management to organizational science, up to the study of individual behaviour and social units, especially in response to a change. Implementation science is contiguous to other fields such as quality improvement, which however usually begins with a specific problem rather than with a new practice to be disseminated [18]. Another adjacent field is that of the traditional dissemination of research results, which however usually focuses on communication, education and information conveyance strategies [19]. Possible errors can occur in both the design and implementation phases knowledge of the implementation context. Furthermore, there is often an expectations gap between program designers, policy makers and implementers. To describe the implementation process, the Theory-Design-Implementation framework remains interesting. The Theory-Design-Implementation framework comprises three main elements: real-world program implementation, the intervention design and the underlying theory of the intervention. Interaction between these elements can be assessed by two indices (implementation index and adaptation index) and two defects (design and implementation) [20]. An ideal intervention aligns optimally with core components of theory both in its design and implementation, but in the real world, design and implementation defects are the norm [21]. In our opinion, an ideal implementation intervention would correspond to squaring the circle, imagining the Intervention Design as a Circle and the Real Word implementation as a Square. The air of the square represents the real implementation, the air of the circle the ideal implementation, while the difference, air in blue, the implement defect (Fig. 2).

Squaring the Circle is an unsolvable problem, but in its design, we ask for the use of two operational tools, i.e., Change Management as a compass and Implementation Science as a straightedge. Change management and implementation science are the perspectives from which to draw operational tools to conduct organizational transformation which finds in the establishment of a community of practice that form of work organization in multidisciplinary teams which the literature has shown to be crucial for improving the outcomes of care and the efficiency of the system. If all stakeholders can and must be involved, the consequence is that their interests must be made mutually transparent in an exercise of social sharing that appears fundamental to making the system robust and durable over time.

It should also be noted that the implementation model for using the CGA guidelines must include specific adaptations based on the application settings. Specifically, the usefulness of adapting the implementation to nine clinical settings categorized into five sections already identified in the PICOs questions (Patient or Population, Intervention, Comparison and Outcome), specifically: Sect. 1. Outpatients in specialist clinics and primary care/general medicine/community: setting (1) general practice and primary care; setting (2) specialist medical outpatient clinics; setting (3) specialist surgical outpatient clinics; Sect. 2. Patients in emergency departments: setting (4) emergency department. Section 3. Patients admitted to hospital: setting (5) hospital medical wards; setting (6) hospital surgical wards; Sect. 4. Patients in long-term care facilities: setting (7) long-term rehabilitation facilities; setting (8) nursing homes and Sect. 5. Patients in hospice and other palliative care facilities: setting (9) Hospice and other palliative care facilities.

It should be underlined that in the analysis of the studies for the current Italian guidelineson CGA for the older persons, no intervention studies were retrieved that investigated the CGA in the hospice and in other palliative care network contexts, and therefore not it was possible to produce clinical practice recommendations for this context [1]. For all outcomes for which data were not available in the literature, in different contexts, the group of experts defined a series of research recommendations, specifically (Table 4), research areas which, as already expressed, appear it is appropriate to typify the implementation interventions.

**Table 4:** Outcomes with research recommendations for different setting

	OUTCOMES							
	Mortality	Hospitalization and/or rehospitalization	Admission to long-term care facilities	Delirium incidence	Prolonged length of stay	Prescriptive appropriateness	Funzional impairment	Emergency department readmission
SETTING								
Section 1 general practice and primary care	No difference CGA vs usual care	Decrease	no difference CGA vs usual care	RR	RR	RR	RR	RR
Section 2 emergency department	RR	Decrease	RR	RR	RR	RR	Decrease	Decrease
Section 3 hospital medical/surgical wards	No difference CGA vs usual care	No difference CGA vs usual care	Decrease	Decrease	RR	RR	RR	RR
Section 4 medical surgical clinics	Decrease	No difference CGA vs usual care	No difference CGA vs usual care	Decrease	Decrease	Increase	RR	RR
Section 5 long term care facilities	No difference CGA vs usual care	No difference CGA vs usual care	RR	RR	RR	RR	RR	RR

*RR* research recommendations

### Future perspectives

The implementation of CGA in older patients imposes a health policy challenge that requires efforts to promote healthy aging, with medical and social obstacles. CGA is a multidimensional diagnostic approach for older adults with the possibility of diagnosis of disease, monitoring and managing it, personalized care and optimization of healthcare resources.

However, remote CGA also has limitations, including technology requirements, data security, and the need for comprehensive evaluation and simplicity. Collaborative efforts are essential to developing a digital CGA platform that addresses accessibility issues and adapts the assessment process to meet the needs of older adults. The integration of CGA with information and communication technologies (ICT) may increase adherence to lifestyle interventions, pharmacological treatments, and in general self-management of chronic diseases that can improve patients’ clinical goals, and reduce negative outcomes [10]. Continuous optimization of digital CGA can become a critical tool to advance geriatric care and ensure the well-being of the older population. Future Projects, such as the recent MULTIPLAT_AGE project, must lead to the product of a coordinated action of research teams with expertise in care transition models, innovative [10]. This vision will lead to allow the development of integrated and coordinated multimodal interventions conveyed by ICT services which can provide multiple healthcare solutions tailored to the needs of patients. This could hopefully improve the quality of care and quality of life among multimorbid older people.

### Concluding remarks

The standardization of CGA in older adults across different settings is particularly important in countries like Italy, that have the oldest world population and where broader implementation of CGA also outside traditional geriatric settings has become a health priority that cannot longer be delayed. Despite decades of CGA experience in clinical and research field [22], where it is decided to assign a central role to the Italian guidelines on CGA for the older persons for the governance of the healthcare system, the consequence that must be drawn is that the interests legitimately involved in the governance of the “life cycle” they are necessarily multiple. No stakeholder should be considered useless in this common and choral effort. If all stakeholders can and must be involved, the consequence is that their interests must be made mutually transparent in an exercise of social sharing which appears fundamental to making the system robust and durable over time. We need to move from the science of CGA to its wide implementation so we can adapt the principles of CGA to local realities with the goal of providing better care across different contexts [23]. In this exercise it appears clear that only the interest of the suffering human person takes on an “absolute” value: social conscience, awareness of contexts and constraints are the cultural pillars on which to build the rest of the “hierarchy” of values.

## Data Availability

The full text of the Guidelines on Comprehensive Geriatric Assessment for Older Person is available in Italian language at the following link: https://www.iss.it/-/valutazione-multidimensionale-persona-anziana-1. No datasets were generated or analysed during the current study.
